# Supplementary material for: Enhancing e-learning through AI: advanced techniques for optimizing student performance
Source: PeerJ Comput Sci. 2024 Dec 23;10:e2576. doi: 10.7717/peerj-cs.2576 (PMC11784796; doi:10.7717/peerj-cs.2576)
Supplement: Supplemental Information 3 [file peerj-cs-10-2576-s003.docx]

# Computing Infrastructure

Operating System
- Windows 11

Hardware
- Processor: Intel Core i7
- RAM: 16GB

 Software Dependencies
- Python 3.8
- pandas
- scikit-learn
- xgboost
- tensorflow

Installation Instructions

To install the necessary dependencies, run the following command:

pip install pandas scikit-learn xgboost tensorflow

Execution

To execute the script, use the following command:

python code.py
