# Supplementary material for: Enhancing e-learning through AI: advanced techniques for optimizing student performance
Source: PeerJ Comput Sci. 2024 Dec 23;10:e2576. doi: 10.7717/peerj-cs.2576 (PMC11784796; doi:10.7717/peerj-cs.2576)
Supplement: Supplemental Information 4 [file peerj-cs-10-2576-s004.docx]

Appendix 1: Abbreviation with descriptions.

| **Abbreviation** | **Description** |
| --- | --- |
|  |  |
| AI | Artificial Intelligence |
| IoT | Internet of Things |
| CNN | Convolutional Neural Network |
| RNN | Recurrent Neural Network |
| LSTM | Long Short-Term Memory |
| ANN | Artificial Neural Network |
| RF | Random Forest |
| DT | Decision Tree |
| XGB | eXtreme Gradient Boosting |
| KNN | K-Nearest Neighbors |
| TP | True Positive |
| TN | True Negative |
| FP | False Positive |
| FN | False Negative |
| LMS | Learning Management System |
| MAE | Mean Absolute Error |
| MSE | Mean Squared Error |
