# Supplementary material for: Enhancing e-learning through AI: advanced techniques for optimizing student performance
Source: PeerJ Comput Sci. 2024 Dec 23;10:e2576. doi: 10.7717/peerj-cs.2576 (PMC11784796; doi:10.7717/peerj-cs.2576)
Supplement: Supplemental Information 5 [file peerj-cs-10-2576-s005.docx]

# README

AI Application for Feature Selection and Model Evaluation

Description

This project evaluates various machine learning and deep learning models using feature selection methods . The models tested include KNN, Decision Tree, Random Forest, XGBoost, CNN, RNN, LSTM, and ANN.

Dataset

The dataset used in this study can be accessed at the following URL:

1-https://www.kaggle.com/datasets/prajwalkanade/student-performance-prediction-dataset

2-https://www.kaggle.com/datasets/mdmahmudulhasansuzan/students-adaptability-level-in-online-education

3-https://www.kaggle.com/datasets/aljarah/xAPI-Edu-Data.

It contains features related to student performance at the university, with the last column being the target variable.

Dependencies

- Python 3.8
- pandas
- scikit-learn
- xgboost
- tensorflow

Install the dependencies using the following command:

pip install pandas scikit-learn xgboost tensorflow

Instructions

1. Download and Prepare the Dataset:
 - Download the dataset from the provided URL.
 - Place the dataset in the same directory as the script.

2. Run the Script:

- Execute the “ code.py ” script using Python 3.8:

 python code.py


3. Feature Selection:
 - The script performs feature selection using Chi-Square and Mutual Information methods.
 - Selected features are used to train and evaluate the models.

4. Model Training and Evaluation:
 - Models are trained on the training dataset and evaluated on the test dataset.
 - Accuracy scores are printed for each model.

 Results

The results are stored in a dictionary and printed at the end of the script. The dictionary contains the model names, feature selection methods, and their corresponding accuracy scores.
